# Identification of transmission foci of *Schistosoma mansoni*: narrowing the intervention target from district to transmission focus in Ethiopia

**DOI:** 10.1186/s12889-020-08904-1

**Published:** 2020-05-24

**Authors:** Abebaw Tiruneh, Daniel Kahase, Endalew Zemene, Eyob Tekalign, Absra Solomon, Zeleke Mekonnen

**Affiliations:** 1 School of Medical Laboratory Sciences, Institute of Health, Jimma University, Jimma, Ethiopia; 2 Department of Medical Laboratory Sciences, College of Medicine and Health Sciences, Wolkite University, Wolkite, Ethiopia; 3 Department of Medical Laboratory Sciences, College of Health Sciences, Mizan-Tepi University, Mizan-Tepi, Ethiopia

**Keywords:** *Schistosoma mansoni*, School-age children, Transmission foci, Ethiopia

## Abstract

**Background:**

*Schistosoma mansoni* (*S. mansoni*) infection is a significant public health problem in Ethiopia, and has wide distribution in the country. The impact of the disease is particularly high on school-age children. Nationwide 385 endemic districts were identified, whereby control and elimination interventions are underway using school-based annual mass drug administration (MDA) with praziquantel. The national elimination program targets endemic districts as a whole. The aim of this study was to identify the transmission foci of *Schistosoma mansoni* and determine prevalence of soil-transmitted helminths (STHs) in Abeshge district.

**Methods:**

The study was conducted from April to May, 2019 among school-age children randomly selected from public elementary schools in Abeshge district, South-central Ethiopia. Demographic information and data on risk factors of *S. mansoni* infection were gathered using pre-tested questionnaire. Moreover, a stool sample was collected from each child and examined using Kato-Katz thick smear technique. The data were analyzed using STATA_MP version 12.

**Results:**

A total of 389 school-age children from five public elementary schools were included in the study. The overall prevalence of *S. mansoni* and STHs was 19.3% (75/389) and 35% (136/389), respectively. The prevalence of *S. mansoni* was 60.6% in Kulit Elementary school, while it was zero in Geraba. The prevalence of *S. mansoni* was significantly higher among males (AOR = 2.6, 95% CI 1.3–5.1), those with habit of swimming and/or bathing in rivers (AOR = 2.9, 95%CI 1.3–5.1) and involved in irrigation activities (AOR = 2.9, 95% CI 1.0–8.3). Overall, the prevalence of *S. mansoni* was significantly higher among school children attending Kulit Elementary School compared to those attending the remaining schools (AOR = 12.5, 95%CI 6.2–25.1).

**Conclusion:**

A wide variation of *S. mansoni* prevalence was observed among the school children in the different schools. Control interventions better identify and target foci of *S. mansoni* transmission, instead of targeting the district homogenously.

## Background

Schistosomiasis (bilharzia) is a parasitic disease caused by blood flukes (trematodes) of the genus *Schistosoma*. Five species of *Schistosoma*, *Schistosoma mansoni* (*S. mansoni*), *Schistosoma japonicum* (*S. japonicum*), *Schistosoma intercalatum* (*S. intercalatum*), *Schistosoma mekongi* (*S. mekongi*) and *Schistosoma haematobium* (*S. haematobium*) are known to cause human schistosomiasis. The intermediate hosts for the two major species, *S. mansoni* and *S. haematobium,* are snails of the genus *Biomphalaria* and *Bulinus*, respectively. The snails inhabit fresh water bodies. The disease is endemic in 78 countries with an estimated 779 million people at-risk and about 190 million people already infected in 2016 [1, 2]. About 90% of the global burden of schistosomiasis occurs in Africa, and is caused by *S. mansoni* (intestinal schistosomiasis) and *S. haematobium* (urogenital schistosomiasis) in the continent [1, 3]. The disease burden is high in sub-Saharan Africa where environmental and host (human) factors favor the transmission and growth of the parasites and their intermediate hosts [4, 5]. The transmission of *Schistosoma* is highly focal; restricted to environments with fresh water bodies infested by appropriate snail intermediate hosts, and where open field defecation and human-water contact is common [6, 7].

The most vulnerable groups to acquire *Schistosoma* infections are school-aged children (SAC) and at-risk adults (pregnant, lactating women and individuals who frequently contact infested fresh water bodies due to their occupation). School-aged children in developing countries are particularly affected by the disease [8]. The administration of preventive chemotherapy during control programs mainly targets this age group. Specific area or localities of the community may fall at different transmission risk levels based on the prevalence of schistosomiasis among SAC [9, 10]. Accordingly, a community may be classified as high risk community (> 50% prevalence), moderate risk community (20–50% prevalence) and low risk community (< 20% prevalence). Based on these categories, mass drug administration (MDA) with praziquantel is recommended to SAC and the whole community once a year in high-risk communities, SAC and at-risk community groups once every 2 years in moderate-risk communities and only SAC twice during their primary schooling in low-risk communities [11, 12].

Ethiopia is among schistosomiasis high-burden countries with estimated 38.3 million people at risk of acquiring the infection and 5.01 million already infected in 2012. The predominant *Schistosoma* species in the country is *S. mansoni*, while *S. haematobium* is mainly limited to areas adjacent to the Awash River Valley [13, 14]. The disease causes devastating health problems including nutritional, cognitive and pathological effects among infected individuals. School-age children particularly suffer growth retardation, low school performance, cognitive impairments, anemia, nutritional disorders and remarkable pathological illness from egg granuloma [10, 15–18]. Considering this, MDA with praziquantel was underway in endemic districts of the country targeting SAC since the last decade [19].

Mapping of schistosomiasis and other Neglected Tropical Diseases (NTDs) at national level was completed recently. The mapping identified 385 endemic districts requiring MDA, and about 5 million SAC were treated with praziquantel in 2015 alone. It is also planned to scale up the MDA coverage from SAC to include at-risk adults for the achievement of the national goal of schistosomiasis control and elimination by 2020 and 2025, respectively [12, 20]. Although substantial successes were recorded in minimizing the disease burden and egg intensity among treated SAC [13, 21], the overall coverage of praziquantel treatment was low (36%) in 2016 [12]. The control program also faced several challenges including incomplete mapping, re-infection, program reliance on MDA and targeting of SAC only [9, 22]. Some of these challenges may partly be due to the classification of *S. mansoni* transmission areas at district level while the transmission is highly focal depending on the existence of fresh water bodies and appropriate snail intermediate hosts. For instance, in a study conducted in Chilga district, Northwest Ethiopia, a wide variation in infection range from zero prevalence in some schools to high burden of infection in other schools was reported [23]. Being in the same district, the MDA is administered in *kebeles* (smallest administrative units/localities in Ethiopia) with no *S. mansoni* transmission, which poses several challenges. This necessitates identifying and targeting at-risk areas where active transmission of *S. mansoni* occur. The aim of this study was to identify the transmission foci of *S. mansoni* and determine prevalence of STHs in Abeshge district.

## Methods

### Study design and setting

A cross-sectional study was conducted from April to May, 2019 in randomly selected public elementary SAC in Abeshge district. The district is located in Gurage zone, 155kms south of Addis Ababa. The weather is warm, and there are rivers in some of the *kebeles*. Small-scale irrigation is practiced in some *kebeles*, which may create favorable conditions for *S. mansoni* transmission. Abeshge district has 25 administrative *kebeles*, with one public elementary school located in each *kebele.* In 2018, the estimated total population of the district was 81,119.

### Sample size and sampling technique

The sample size was calculated as 400 using single population proportion formula, assuming proportion of infection among the children to be 0.13 [24], at 95 confidence level and margin of error of 0.05, design effect of 2 and 15% non-response rate.

First, five elementary schools were randomly selected from the total of 25 primary schools located in the district. The selected schools were Geraba, Kulit, Jeju, Walga and Hole elementary schools and the total number of SAC in each school was 651, 595, 643, 635 and 691, respectively. The total sample size was allocated to the selected schools proportional to the total number of the SAC in each school. Systematic random sampling technique was used to select the study participants from each class room using class rosters as sampling frame. Accordingly, 80, 86, 74, 79 and 81 SAC were selected from Jeju, Hole, Kulit, Walga and Geraba Elementary Schools, respectively. School children who were treated with anti-helminthic drugs in the last 2 weeks prior to the survey and those who could not provide stool samples after two visits were excluded from the study. Few of the SAC were 15 years of age, likely due to late school enrolment.

### Data collection

Semi-structured questionnaire was used to collect data on socio-demographic and associated risk factors of schistosomiasis among the SAC. The questionnaire was first developed in English and translated to Amharic (Additional file 1). Three trained laboratory personnel interviewed parents/guardians of the children after obtaining written consent. Moreover, thumb-sized stool sample was collected from each child using clean leak-proof stool cups. School-aged children who could not provide stool sample on the first visit were instructed to provide on the following day. The collected stool samples were transported to Wolkite University Microbiology and Parasitology Laboratory using cold box. Kato-Katz thick smear technique was employed for the detection and quantification of *S. mansoni* and STH eggs, following standard operating procedures. A single specimen of each child and single Kato-Katz smear of each specimen was processed.

### Data analysis

Egg per gram (EPG) of *S. mansoni* was obtained by multiplying the number of eggs counted in 41.7 mg of the Kato-Katz template by the factor 24 [25]. The infection intensity was classified as light (1–99 EPG), moderate (100–399 EPG) and heavy (> 400 EPG) infection [11]. The data were entered into Microsoft Excel and cleaned. The data were analyzed using STATA_MP version 12 (Stata Corp., TX, USA). Descriptive statistics including frequency, percentage and mean were computed to summarize socio-demographic characteristics of the SAC. Bivariate analysis of each independent variable with the dependent variable was performed. Variables with *p* value less than 0.2 during bivariate analysis were candidates for the multivariable analysis. Odds ratios and the corresponding 95% confidence intervals were calculated to measure the strength of association. Statistical significance was set at *p* value less than 0.05.

## Results

### Demographic characteristics of the study participants

A total of 389 SAC were enrolled in this study with response rate of 97%. Out of the total study participants, 199 (51.2%) were males and 218(56%) were in the age group of 5–10 years. Thirty-eight (9.8%) of the study participants were from households lacking their own latrine, and about half (50.4%) practiced open field defecation. Majority of family members of the study participants (91.3%) obtain drinking water from protected sources (Table 1).

**Table 1:** Socio-demographic characteristics of the study participants, Abeshge district, South-central Ethiopia

Characteristics		Frequency n(%)
Age group	5–10	218(56.0)
	11–15	171(44.0)
Sex	Male	199 (51.2)
	Female	190 (48.8)
Grade	1–4	297 (76.3)
	5–8	92 (23.7)
Family size	< 5	121 (31.1)
	> 5	268 (68.9)
Occupation of household head	Farmer	275 (70.7)
	Merchant	65 (16.7)
	Daily laborer	22 (5.7)
	Employed	27 (6.9)
Latrine availability	No	38 (9.8)
	Yes	351 (90.2)
Latrine use	Sometimes	196 (50.4)
	Always	193 (49.6)
Drinking water source	Protected	355 (91.3)
	Unprotected	34 (8.7)

## Prevalence of *Schistosoma mansoni* and STHs

The overall prevalence of *S. mansoni* and STHs was 19.3% (75/389) and 35% (136/389), respectively. Other intestinal parasites including *Enterobius vermicularis* (*E. vermicularis*), *Hymenolepis nana* (*H. nana*) and *Taenia* species were detected in less than 5% of the study participants. All the STH infections were due to the hookworms (except a single case of *Trichuris trichiura*). The geometric mean egg count of *S. mansoni* was 191.3 EPG. Light, moderate and heavy infections of *S. mansoni* were 28(37.3%), 26(34.7%) and 21(28%), respectively. None of the SAC from Geraba Elementary School were positive for *S. mansoni*, whereas 60.6% (43/71) of the children from Kulit Elementary School were *S. mansoni* infected (Table 2).

**Table 2:** Prevalence of *Schistosoma mansoni* among each elementary school children, Abeshge district, South-central Ethiopia

Name of the Elementary School	# examined (%)	***Schistosoma mansoni*** infection	
		Positive n(%)	Negative n(%)
Jeju	80 (20.6)	7 (8.8)	73 (91.2)
Hole	86 (22.1)	10 (11.6)	76 (88.4)
Kulit	71 (18.3)	43 (60.6)	28 (39.4)
Walga	76 (19.5)	16 (21.1)	60 (78.9)
Geraba	76 (19.5)	0 (0)	76 (100)

### Risk factors associated with *Schistosoma mansoni* infection

Out of the total study participants, 205(52.7%) had the habit of swimming and/or bathing in rivers. The prevalence of *S. mansoni* among SAC who had the habit of swimming and/or bathing in rivers was 29.8% (61/205). School-age children who responded participation in irrigation activities were 28(7.2%), half of whom were positive for *S. mansoni*. Although latrine coverage among households of the study participants was 90.2%, open field defecation habit was reported by 50.4% of the SAC. Using bivariate analysis (Table 3), sex (COR = 2.6, 95%CI 1.5–4.4), habit of swimming and/or bathing in rivers (COR = 1.9, 95%CI 1.1–3.3), participation in irrigation activities (COR = 4.9, 95%CI 2.2–10.8) and residence/school (COR = 13.7, 95%CI 7.5–25.0) were significantly associated with *S. mansoni* infection.

**Table 3:** Bivariate analysis of factors associated with *Schistosoma mansoni* infection, Abeshge district, South-central Ethiopia

Variables		Examined n(%)	Positive n(%)	COR(95%CI)	***p***-value
Age group	5–10	218(56.0)	39 (17.9)	Ref	Ref
	11–15	171(44.0)	36 (21.1)	1.2 (0.7–2.0)	0.433
Sex	Male	199 (51.2)	52 (26.1)	2.6 (1.5–4.4)*	0.001
	Female	190 (48.8)	23 (12.1)	Ref	Ref
Grade	1–4	297 (76.3)	62 (20.9)	1.6 (0.8–3.1)	0.155
	5–8	92 (23.7)	13 (14.1)	Ref	Ref
Family size	< 5	121 (31.1)	26 (21.5)	1.2 (0.7–2.1)	0.459
	> 5	268 (68.9)	49 (18.3)	Ref	Ref
Household head occupation	Merchant	65 (16.7)	10 (15.4)	Ref	Ref
	Farmer	275 (70.7)	54 (19.6)	1.3 (0.6–2.8)	0.432
	Daily laborer	22 (5.7)	6 (27.3)	2.1 (0.6–6.5)	0.219
	Employed	27 (6.9)	5 (18.5)	1.3 (0.4–4.1)	0.711
Latrine availability	No	38 (9.8)	8 (21.1)	1.1 (0.5–2.6)	0.771
	Yes	351 (90.2)	67 (19.1)	Ref	Ref
Latrine use	Sometimes	196 (50.4)	38 (19.4)	1.0 (0.6–1.7)	0.957
	Always	193 (49.6)	37 (19.2)	Ref	Ref
Drinking water source	Protected	355 (91.3)	68 (19.2)	Ref	Ref
	Unprotected	34 (8.7)	7 (20.6)	1.1 (0.5–2.6)	0.840
Swimming and/or bath in rivers	No	184 (47.3)	14 (7.6)	Ref	Ref
	Yes	205 (52.7)	61 (29.8)	5.1 (2.8–9.6)*	0.001
Washing cloth in rivers	No	107 (27.5)	18 (16.8)	Ref	Ref
	Yes	282 (72.5)	57 (20.2)	1.3 (0.7–2.2)	0.450
Participation in irrigation	No	361 (92.8)	61 (16.9)	Ref	Ref
	Yes	28 (7.2)	14 (50.0)	4.9 (2.2–10.8)*	0.001
School	Kulit	71 (18.3)	43 (60.6)	13.7 (7.5–25.0)*	0.001
	Others	318 (81.7)	32 (10.1)	Ref	Ref

*Ref* Reference *Statistically significant at *p* < 0.05

Predictors of *S. mansoni* infection are presented in Table 4. Accordingly, after adjusting for other variables, there were significant differences in prevalence of *S. mansoni* by sex (AOR = 2.6, 95% CI 1.3–5.1), the habit of swimming and/or bathing in rivers (AOR = 2.9, 95%CI 1.3–5.1), involvement in irrigation (AOR = 2.9, 95% CI 1.0–8.3) and attending Kulit Elementary School (AOR = 12.5, 95%CI 6.2–25.1).

**Table 4:** Multivariable analyses of risk factors of *Schistosoma mansoni* infection, Abeshge district, South-central Ethiopia

Characteristics		Examined n(%)	Positive (%)	AOR(95%CI)	***p***-value
Age group	5–10	218(56.0)	39 (17.9)	Ref	Ref
	11–15	171(44.0)	36 (21.1)	1.3 (0.6–2.6)	0.542
Sex	Male	199 (51.2)	52 (26.1)	2.6 (1.3–5.1)	0.001*
	Female	190 (48.8)	23 (12.1)	Ref	Ref
Grade	1–4	297 (76.3)	62 (20.9)	1.7 (0.7–4.1)	0.267
	5–8	92 (23.7)	13 (14.1)	Ref	Ref
Household head occupation	Merchant	65 (16.7)	10 (15.4)	Ref	Ref
	Farmer	275 (70.7)	54 (19.6)	1.7 (0.7–4.2)	0.233
	Daily laborer	22 (5.7)	6 (27.3)	3.5 (0.9–14.0)	0.075
	Employed	27 (6.9)	5 (18.5)	0.9 (0.2–4.1)	0.927
Water source	Protected	355 (91.3)	68 (19.2)	ref	Ref
	Unprotected	34 (8.7)	7 (20.6)	0.9 (0.3–2.6)	0.829
Swimming and/or bath in rivers	No	184 (47.3)	14 (7.6)	Ref	Ref
	Yes	205 (52.7)	61 (29.8)	2.9 (1.3–5.1)	0.004*
Washing clothes in river	No	107 (27.5)	18 (16.8)	Ref	Ref
	Yes	282 (72.5)	57 (20.2)	1.2 (0.6–2.3)	0.653
Participate in irrigation activities	No	361 (92.8)	61 (16.9)	Ref	Ref
	Yes	28 (7.2)	14 (50.0)	2.9 (1.0–8.3)	0.046*
Schools	Kulit	71 (18.3)	43 (60.6)	12.5 (6.2–25.1)	0.001*
	Others	318 (81.7)	32 (10.1)	Ref	Ref

*Ref* Reference *Statistically significant at *p* < 0.05

## Discussion

This study aimed at identifying transmission foci of *S. mansoni* by determining its prevalence among SAC in the district. Overall, nearly a fifth of the SAC had *S. mansoni* infection and more than a third of the children had STH infections. Almost all the STH infections were caused by hookworms. Hookworm infections may result in iron deficiency anemia, adversely affecting school performance of the children [26]. The prevalence of both *S. mansoni* and STHs was higher compared to the earlier report from the same district [24]. The difference could be due to wide variation in prevalence of *S. mansoni* infection in the different sampled *kebeles*. In spite of school MDA interventions [19], transmission of the disease is ongoing. Depending on how long the children were infected, the children may experience clinical manifestations including acute Katayama fever and chronic egg granuloma which may result in portal hypertension and gastro-intestinal bleeding [27]. This may lead to malnutrition and ill health with subsequent impact on school performance and absenteeism [17, 18, 28, 29]. The disease may also adversely affect productivity of adults in the district as the disability adjusted life years (DALYs) due to schistosomiasis is high in Africa [10, 30].

Human infection with *S. mansoni* occurs by skin penetration with cercaria in fresh water bodies. In this study, children who reported to swim and/or bath in rivers were three times more likely to be infected with *S. mansoni* as compared to those who did not have the habit. Studies conducted elsewhere also revealed significant association of contact with fresh water bodies and *S. mansoni* infection [31–33]. On the other hand, half of the study participants practice open field defecation which may result in fecal contamination of water bodies in the area. The prevalence of *S. mansoni* was relatively higher in Kulit and Walga *kebeles*. The presence of rivers crossing these areas may favor transmission of *S. mansoni* in those *kebeles*. Moreover, small-scale irrigation is common in these *kebeles*. Children who reported participation in irrigation activities had significantly higher prevalence of *S. mansoni* infection compared to those who did not participate in irrigation. The construction of hydro-electric dams and irrigation projects in Ethiopia may create favorable habitat for snail intermediate hosts [34]. For instance, the incidence of *S. mansoni* was increased following the construction of irrigation microdams in Tigray region, Northern Ethiopia [35]. The abundance of *Biomphalaria pfeifferi* also ascend from the source river to secondary (line from river) and tertiary (line to/in the farm) irrigation canals [36] which may increase the likelihood of cercaria skin penetration during irrigation activities. Other studies also reported the risk of irrigation to *S. mansoni* infection among children and adults [31, 37].

In this study*, S. mansoni* infection was significantly associated with sex of the children, with males more than twice likely to be infected compared to females. This could be attributed to difference in behavior of males compared to the females. Males often play outdoors and the frequency of contact with river water is likely higher. In terms of social-cultural context, males have freedom to swim and bath in rivers compared to the females, thereby predisposing the males to higher risk of *S. mansoni* infection [38, 39]. Similarly, studies done in Ejaji [40] and Wolaita [41] also reported significantly higher prevalence of *S. mansoni* infection among male children compared to females.

Parasitic infections often have focal distribution, depending on several environmental and social factors. In this study, SAC from Kulit had significantly higher prevalence of *S. mansoni* infection compared to the other schools. This is likely due to presence of river crossing the *kebele* and presence of small-scale irrigation. Considering prevalence heterogeneity among *kebeles* within the same district is important during schistosomiasis control and elimination interventions. The Ethiopian NTDs master plan classified 48.6% (385/793)*,* 44.8% (355/793) and 6.7% (53/793) of the districts of the country as *Schistosoma*-endemic, *Schistosoma*-free and districts with unknown prevalence, respectively. The *Schistosoma*-endemic districts require preventive chemotherapy [20]. However, due to the high focal transmission nature of the disease [7], classification of transmission intensity at district level may result in unnecessary treatment of *Schistosoma*-free *kebeles* in endemic districts. *S. mansoni* control and elimination interventions should be based on fine-scale mapping within districts [42]. In this study, the prevalence of *S. mansoni* ranged from zero (Geraba elementary school) to 60.6% (Kulit elementary school), which indicates a wide variation in transmission intensity within the same district. The annual MDA given for the SAC is also similar between low to high prevalence *kebeles*. Earlier study conducted among SAC in Chilga district, Northwest Ethiopia also documented three out of seven elementary schools had zero prevalence of *S. mansoni* [23]. This calls for locality-specific intervention program in the same district by considering transmission intensity/prevalence variations. The national health system and health sector transformation plan also support district-based disease assessment and control interventions to assess disease profile of each *kebele* [43].

Narrowing the target area from district to transmission foci will also help in resolving challenges of the current national program including coverage query, its dependence on school-based MDA, and high re-infection rate of *S. mansoni* particularly in high transmission settings [7, 9, 22]. The coverage of MDA among SAC in Ethiopia is still less than half of the plan and about 53 districts lack baseline prevalence to classify the level of endemicity of schistosomiasis [9, 20]. It also appears that less emphasis is given to snail control and environmental management at national level. However, interventions targeting the snail intermediate hosts remarkably help in the control and elimination of the disease [44]. Providing annual MDA for SAC in Ethiopia may not address other age groups and SAC who are out of school. The burden of *S. mansoni* in this study may also indicate high burden of schistosomiasis among their family members [45]. This may sustain the transmission by provoking environmental fecal contamination and fuel re-infection rate after MDA.

In this study, the single stool specimen of each child and single Kato-Katz smear of each specimen processed might have underestimated the prevalence. Moreover, identification of the transmission foci and burden of infection in this study involved only stool examination with no malacological component, which could provide additional information on the transmission [46–48].

## Conclusion

A wide variation in the prevalence of *S. mansoni* was observed among the different schools. *S. mansoni* infection was significantly higher among males, children who had habit of swimming and/or bathing and in those participating in irrigation activities. Therefore, health information on the risk factors and prevention methods of schistosomiasis need to be provided for the SAC. Schistosomiasis control and elimination interventions better identify and target foci of *S. mansoni* transmission instead of targeting the district homogenously.

## Supplementary information

**Additional file 1 **(A) Questionnaire developed for the assessment of socio-demographic characteristics of the school children. (B) Questionnaire developed to assess risk factors of *S. mansoni* infection among the school children

## Data Availability

All materials and data are available in the hands of corresponding author if requested.

## Abbreviations

DALYs: Disability-adjusted life years
EPG: Eggs per gram
MDA: Mass drug administration
NTDs: Neglected tropical diseases
SAC: School-age children
STHs: Soil-transmitted helminths
WHO: World health organization
