# Supplementary material for: Identification of transmission foci of Schistosoma mansoni: narrowing the intervention target from district to transmission focus in Ethiopia
Source: BMC Public Health. 2020 May 24;20:769. doi: 10.1186/s12889-020-08904-1 (PMC7245888; doi:10.1186/s12889-020-08904-1)
Supplement: Supplementary file 1 — Additional file 1 (A) Questionnaire developed for the assessment of socio-demographic characteristics of the school children. (B) Questionnaire developed to assess risk factors of S. mansoni infection among the school children [file 12889_2020_8904_MOESM1_ESM.docx]

Additional file 1

1. Questionnaire developed for assessment of socio-demographic characteristics of the school children

| **Q.no** | **Questions** | **Response** | **Code** |
| --- | --- | --- | --- |
| A1 | Age of the child (completed years) | ___years |  |
| A2 | Family size | ____ |  |
| A3 | Sex of the child | 1. Male 2. Female |  |
| A4 | What is the major occupation of the household head? | 1. Farmer 2. Merchant 3. Employed 4. Daily laborer |  |
| A5 | Indicate name of the elementary school your child is attending? | 1. Jeju 2. Hole 3. Kulit 4. Walga 5. Geraba |  |
| A6 | What grade is the child attending? | _____grade |  |
| A7 | Do you have latrine? | 1. Yes 2. No |  |
| A8 | If you have latrine, how often does the child use it? | 1. Sometimes 2. Always |  |

1. Questionnaire developed to assess risk factors of *S. mansoni* infection among the school children

| **Q. no** | **Questions** | **Response** | **Code** |
| --- | --- | --- | --- |
| B1 | Drinking water source | 1. Protected 2. Not protected |  |
| B2 | Does the child swim/bath in river? | 1. Yes 2. No |  |
| B3 | Does the child wash clothes in river? | 1. Yes 2. No |  |
| B4 | Does the child participate in irrigation activities? | 1. Yes 2. No |  |
